# *In vitro* and *in vivo* anti-herpes simplex virus activity of monogalactosyl diacylglyceride from *Coccomyxa sp*. *KJ* (IPOD FERM BP-22254), a green microalga

**DOI:** 10.1371/journal.pone.0219305

**Published:** 2019-07-16

**Authors:** Kyoko Hayashi, Jung-Bum Lee, Kinya Atsumi, Mana Kanazashi, Tamaki Shibayama, Kazumasa Okamoto, Toshio Kawahara, Toshimitsu Hayashi

**Affiliations:** 1 College of Engineering, Chubu University, Kasugai, Aichi, Japan; 2 Graduate School of Medicine and Pharmaceutical Sciences for Research, University of Toyama, Toyama, Toyama, Japan; 3 DENSO CORPORATION, Kariya, Aichi, Japan; 4 Faculty of Engineering, Hokkaido University, Sapporo, Hokkaido, Japan; 5 The Institute of Scientific and Industrial Research, Osaka University, Ibaraki, Osaka, Japan; Cornell University, UNITED STATES

## Abstract

A monogalactosyl diacylglyceride (MGDG) was isolated as an antiviral component from *Coccomyxa* sp. KJ (IPOD FERM BP-22254) *via* bioassay-guided fractionation. α-Linolenic acid (C18:3) and 7,10,13-hexadecatrienoic acid (C16:3) accounted for approximately 72% and 23%, respectively, of the MGDG total fatty acids of the MGDG. The MGDG showed virucidal activity against herpes simplex virus type 2 (HSV-2), a pathogen that causes genital herpes. Physical changes in HSV-2 shape were observed after treatment with MGDG, including a decrease in particle size, and possible damage to the viral envelope, as assessed using electron microscopy. In accordance with the morphological findings, virus particles lost their ability to bind to host cells. HSV-2 treated with high concentrations of MGDG resulted in no pathogenicity in an animal model, indicating that MGDG exhibits irreversible virucidal activity against HSV-2 particles. In the animal model of HSV-2-induced genital herpes, intravaginally administered MGDG exerted a prophylactic effect by suppressing viral yields in the genital cavity and formation of herpetic lesions, resulting in a higher survival rate in treated mice than control mice administered solvent. Thus, MGDG offers a novel prophylactic option against HSV infections.

## Introduction

Herpes simplex virus types 1 (HSV-1) and 2 (HSV-2) primarily infect epithelial tissues before spreading to the nervous system, causing lifelong infection in the sacral ganglia. Genital herpes caused by HSV is a common sexually transmitted infection among populations worldwide [1,2]. Primary and recurrent infections of HSV are often asymptomatic or of short duration in most immunocompetent individuals; however, some immunocompromised individuals, particularly elderly people, transplant patients, and acquired immune deficiency syndrome (AIDS) patients, experience severe primary diseases and frequent symptomatic recurrences [3,4]. Current standard treatments for HSV infections include synthesized nucleoside analog antiviral drugs, such as acyclovir (ACV), famciclovir, and valacyclovir, those are pro-drugs that are activated by viral thymidine kinase to eventually produce triphosphate form [5–8]. HSV strains that are resistant to nucleoside analogs, including ACV, have emerged [9], resulting in a need for development of novel anti-herpes drugs with a different mode of action from that of the nucleoside analogs. Especially, it is desirable to develop agents with virucidal activity to minimize the emergence of drug-resistant mutants.

*Coccomyxa* sp. KJ (IPOD FERM BP-22254) is a green microalga that accumulates lipids at more than 30% dry weight under suitable conditions; therefore, it has received interest as a source for biofuel production [10,11]. Recently, we used bioactivity-guided assays to search for antiviral components from *Coccomyxa* sp. KJ. As a result, a monogalactosyl diacylglyceride (MGDG) was found to have anti-HSV properties.

To date, reports have described the bioactivity of MGDGs from marine products. For example, MGDGs from the green alga *Chlorella vulgaris* [12] reportedly have anti-tumor promoting properties, and MGDGs from the marine sponge *Phyllospongia foliascens* [13], blue green alga [14], and edible brown seaweed [15] exert anti-inflammatory activity. MGDGs isolated from spinach leaves and *Citrus hystrix* have also been shown to inhibit Epstein-Barr virus activation [16,17]. Recently, MGDGs with unknown chemical structures isolated from *Clinacanthus nutans*, a Thai herbal medicine, reportedly have anti-HSV activity *in vitro* [18].

In this study, we assessed the virucidal activity of MGDG, isolated from *Coccomyxa* sp. KJ, for its virucidal activity against HSV-2, and further elucidated its mechanism of action.

## Materials and methods

### Fractionation and chemical structure of MGDG

*Coccomyxa* sp. KJ isolated from a hot spring was grown in an inorganic medium as described elsewhere [10]. The isolation of MGDG from the EtOH extract of dried alga was performed as follows: the EtOH extract was applied to a Diaion HP-20 column chromatography (3.5 × 57 cm) and successively eluted with H_2_O, 50% EtOH, EtOH, and acetone. Each fraction was concentrated under reduced pressure (DE1, 400.9 mg; DE2, 310.3 mg; DE3, 2.25 g; and DE4 4.86 g). The acetone fraction (DE4) was subjected to column chromatography on a silica gel (3 × 42 cm). The column was successively eluted with hexane, hexane-AcOEt (1:1), AcOEt, AcOEt-acetone (1:1), acetone, and MeOH to give DE4A (55.4 mg), DE4B (2.0 g), DE4C (89.0 mg), DE4D (1.01 g), DE4E (95.5 mg), and DE4F (177.1 mg), respectively. DE4D was subjected to silica gel column chromatography (1.5 × 35 cm) using a solvent system of AcOEt-acetone to give three fractions, DE4D1 (49.9 mg), DE4D2 (705.1 mg), and DE4D3 (16.2 mg). DE4D2 was subjected to silica gel column chromatography (1.5 × 35 cm) and eluted with CHCl_3_-MeOH-H_2_O (10:1:0.1) to give DE4D2A (15.6 mg) and DE4D2B (686.5 mg). The later fraction was then subjected to LH-20 column chromatography using a solvent system of CHCl_3_-MeOH (3:1), resulting in DE4D2B1 (11.6 mg) and DE4D2B2 (652.3 mg). DE4D2B2 was applied to silica gel column chromatography (2 × 50 cm) and eluted with CHCl_3_, CHCl_3_-MeOH (20:1), and CHCl_3_-MeOH-H_2_O (10:3:1) to give four fractions, DE4D2B2A (4.8 mg), DE4D2B2B (3.5 mg), DE4D2B2C (25.1 mg), and DE4D2B2D (620.5 mg). Finally, DE4D2B2D was purified on preparative thin layer chromatography (PLC) developed with (CHCl_3_-MeOH-AcOH-H_2_O = 80:9:12:2) to give MGDG (578.9 mg). MGDG was dissolved in MeOH, and acetyl chloride was added and incubated at 50°C overnight. The solution was dried under N_2_, and the residue was analyzed using gas chromatography–mass spectrometry (GC-MS) after reconstitution in hexane. GC-MS analysis was performed on a Shimadzu QP-5000 apparatus equipped with a DB-1 capillary (0.32 × 30 cm). Oven temperature was set at 50°C for 2 min, then vamped at 40°C/min until 270°C. Injector temperature and interface temperature were set at 240°C and 250°C, respectively. Nuclear magnetic resonance (NMR) spectra of MGDG were identical to reported spectra (S1 Fig).

### Cells and viruses

Vero (African green monkey kidney) cells were grown in Eagle’s minimum essential medium (MEM) supplemented with 5% fetal bovine serum (FBS). HSV-1 strain KOS and HSV-2 strain UW 268 were propagated in Vero cells grown in MEM supplemented with 2% FBS at 37°C and stored at -80°C until use.

### *In vitro* antiviral assay

Samples were dissolved in dimethyl sulfoxide at concentrations less than 0.5%, which did not interfere with the growth of cells or viruses. A cytotoxic assay was performed on uninfected subconfluent Vero cells by adding samples to the cell monolayers. Viable cells were counted using the trypan blue exclusion test after 72 h at 37°C. Cytotoxicity was determined by calculating the 50% cytotoxic concentration (CC_50_) from concentration-response curves. In the antiviral assays, confluent Vero cell monolayers were infected at 0.1 plaque-forming units (PFU)/cell for 1 h at room temperature, washed with phosphate-buffered saline (PBS), and then incubated at 37°C for 24 h. Samples were added during virus infection and throughout the incubation thereafter (experiment A) or immediately after virus infection (experiment B). Virus yields were determined using a plaque assay on Vero cell monolayers. The 50% effective concentration (EC_50_) was obtained from the concentration-response curves. Antiviral activities were estimated by the selectivity index calculated from the CC_50_s and EC_50_s. Data are expressed as means ± SD from duplicate assays.

### Time-of-addition experiments

Vero cell monolayers were infected with HSV-2 at 10 PFU/cell for 1 h at room temperature. MGDG was added to the culture medium at concentrations of 25 or 50 μg/ml 3 h before viral infection, during infection for 1 h, and throughout the incubation thereafter, immediately after infection, at 1 h post-infection (p.i.), at 3 h p.i., or at 6 h p.i. The cell cultures were harvested at 24 h p.i. and subjected to a plaque assay.

### Virucidal assay

To determine the effect of MGDG on direct inactivation of virus particles, HSV-2 (2 × 10^5^ PFU/ml) was treated with an equal volume of the compound at a final concentration of 0.01, 0.1, 1, 10, and 50 μg/ml. After 0, 5, 10, 30, 60, 120, 180, and 360 min at 37°C, 100-fold dilutions of the mixture were added to Vero cell monolayers for 1 h at room temperature to be plaque-titrated. The plaque number at 0 h was taken as 100%.

### Virus binding assay

In order to evaluate whether MGDG could lyse HSV-2 envelope, first, the binding ability of MGDG-treated virus to host cell membrane was estimated. Virus (2 × 10^3^ PFU/ml) was treated with an equal volume of MGDG at a final concentration of 0 μg/ml or 50 μg/ml at 37°C for 30 min and then cooled down to 4°C for 1 h. Confluent Vero cell monolayer pre-cooled at 4°C for 3 h were infected with the MGDG-treated virus (100 PFU/well) at 4°C for 1 h, washed 3 times with ice-cold PBS, and then incubated at 37°C for 3 days to be plaque-assayed.

### Cell pretreatment assay

To evaluate whether pre-treatment of cells with MGDG would result in resistance to infection, confluent Vero cell monolayers were incubated at 37°C for 0, 1, 3 and 6 h in the absence or presence of 50 μg/ml MGDG, washed 3 times with PBS, and then infected with HSV-2 (100 PFU/well) for 1 h at room temperature. After washing with PBS, the cell monolayers were subjected to plaque assay.

### Transmission electron microscopy (TEM)

Vero cells were infected with HSV-2 at 5 PFU/cell and incubated in FBS-free MEM at 37°C for 20 h. The medium was harvested and centrifuged at 3,000 rpm for 10 min at 4°C. The supernatants were treated with PBS or 50 μg/ml MGDG for 30 min, fixed for 30 min at room temperature in PBS with 5% glutaraldehyde, and attached to grids. Samples were observed with a JEM ARM 1300 high-voltage electron microscope (JEOL, Japan), and the particle diameter distribution was measured. In another experiment, in order to compare the shape of virus particles released from MGDG-treated cells with those released from untreated cells, Vero cell monolayers were treated with or without 50 μg/ml MGDG at 37°C for 3 h, infected with HSV-2 at 5 PFU/cell and incubated in FBS-free MEM for 20 h. The released viruses were subjected to negative staining electron microscopic analysis.

### *In vivo* animal experiments

Female BALB/c mice (5–6 weeks old) were purchased from Japan SLC (Shizuoka, Japan). All experiments were conducted in accordance with the animal experimentation guidelines of Chubu University and approved by the Animal Care Committee at Chubu University. To increase their susceptibility to vaginal HSV infection [19], mice were subcutaneously injected with medroxyprogesterone 17-acetate at a dose of 3 mg/mouse on days 6 and 1 before virus inoculation. During the initial experiments, we evaluated the irreversibility of virucidal activity of MGDG in a murine model. The virus (2 × 10^4^ PFU) was inoculated with MGDG or its diluent (PBS) for 30 min at room temperature and a final drug concentration of 0, 10, 50, or 250 μg/ml and then inoculated into mice (n = 5). During the second experiments, we investigated the protective effects of MGDG in a genital herpes murine model. Mice were inoculated vaginally with 2 × 10^4^ PFU HSV-2. MGDG (0, 0.2, or 1 mg/day) was administered intravaginally twice per day at a volume of 20 μl PBS containing the compound starting from 1 h before virus inoculation until 7 days after inoculation (n = 5). ACV was also applied topically twice a day at a dose of 0.2 mg per day. Clinical signs of infection were graded according to a six-point scale: 0, no sign of infection; 1, slight genital erythema and edema; 2, moderate genital inflammation; 3, severe exudative genital lesions; 4, hind limb paralysis; and 5, death. The surviving animals were monitored every day at 9 and 18 o’clock for 14 days after virus inoculation. The animals showing clinical sign of infection more than 2 were weighted daily during the experiments. All mice with hind-limb paralysis and decreased body weight more than 20% during one day were euthanized within 24 h (experimental end point) by anesthesia. Viral shedding was determined at day 3 after virus infection by washing the vaginal cavity with 100 μl PBS and titrating the virus using a plaque assay on Vero cell monolayers.

### Statistical analysis

Data except for animal studies were analyzed for statistical significance by Student’s *t-*test or one-way ANOVA and then by post-hoc analysis using Dunnett’s test. In the animal studies, lesion scores and survival rates were analyzed using a Mann-Whitney U test and log-rank test, respectively.

## Result

### Isolation and structural analysis of MGDG

**Fig 1:**
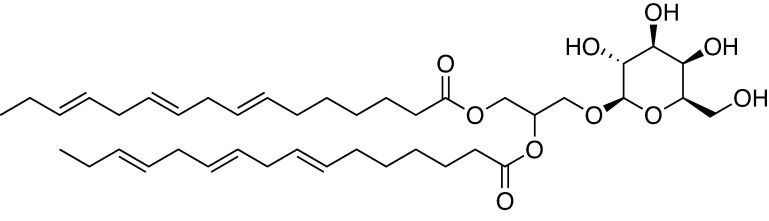
Chemical structure of the most abundant monogalactosyl diacylglyceride (MGDG) occurring in Coccomyxa sp. KJ.

MGDG was isolated from the ethanol extract of dried material as an anti-HSV-2 component contained in *Coccomyxa* sp. KJ. The fatty acid composition pattern of the isolated MGDG is shown in Table 1. α-Linolenic acid (C18:3) and 7,10,13-hexadecatrienoic acid (C16:3) accounted for approximately 72% and 23%, respectively, of the total MGDG fatty acids. The chemical structure of the most abundant MGDG is shown in Fig 1.

**Table 1:** Composition of fatty acids from monogalactosyl diacylglyceride (MGDG) from *Coccomyxa* sp. KJ.

Identification	mol %
Myristic acid (C14:0)	0.13
7,10,13-Hexadecatrienoic acid (C16:3)	22.62
Palmitic acid (C16:0)	1.59
α-Linolenic acid (C18:3)	71.95
Oleic acid (C18:1)	2.70
Stearic acid (C18:0)	1.01

### *In vitro* antiviral effects of MGDG

The effects of *Coccomyxa* sp. KJ ethanol extracts, as well as low and high molecular fractions of a hot water extract prepared from dried materials were evaluated for *in vitro* inhibition of HSV-2 replication (Table 2). The selectivity index of the ethanol extract was more than 10, indicating potential as an antiviral material. Both fractions of the hot water extract showed relatively lower anti-HSV-2 selectivity.

**Table 2:** Anti-herpes simplex virus (HSV)-2 activities of crude extracts prepared from dried material of *Coccomyxa* sp. KJ.

Sample	Cytotoxicity	Antiviral activity (EC_50_, μg/ml)		Selectivity index (CC_50_/EC_50_)	
	(CC_50_, μg/ml)	A^a^	B^b^	A	B
EtOH extract	830 ± 71	76 ± 5.7	110 ± 14	11 ± 0.71	7.6 ± 1.1
LW^c^	370 ± 14	57 ± 16	66 ± 9.2	6.8 ± 2.1	5.7 ± 0.99
HW^d^	1500 ± 71	340 ± 35	400 ± 71	4.4 ± 0.21	3.7 ± 0.14

Each value is mean ± SD from independent duplicate assays.

^a^ Sample was added during viral infection and throughout the incubation thereafter.

^b^ Sample was added immediately after viral infection.

^c^ Low molecular weight (LW) fraction of hot water extract.

^d^ High molecular weight (HW) fraction of hot water extract.

MGDG was isolated as an active component contained in the ethanol extract after antiviral assay-guided fractionation. As shown in Table 3, MGDG showed antiviral activities against both HSV-1 and HSV-2, with similar selectivity indices. There were no marked differences in antiviral activity between treatments in which MGDG was present in the medium during virus infection and throughout the incubation thereafter (experiment A) or treatments in which MGDG was added immediately after virus infection (experiment B). The effect of MGDG on viral attachment to host cells and the entry of virus into cells in part could be evaluated in experiment A but not in experiment B, suggesting that MGDG might not interfere with the early stages of virus infection, including the virus-binding step.

**Table 3:** Anti-herpes simplex virus (HSV)-1 and anti-HSV-2 activities of monogalactosyl diacylglyceride (MGDG).

Virus	Cytotoxicity	Antiviral activity (EC_50_, μg/ml)		Selectivity index (CC_50_/EC_50_)	
	(CC_50_, μg/ml)	A^a^	B^b^	A	B
HSV-1	150 ± 3.5	12 ± 0.42	14 ± 0.28	11 ± 0.49	11 ± 0.49
HSV-2	150 ± 3.5	11 ± 0.42	11 ± 1.6	14 ± 0.92	13 ± 2.1

Each value is the mean ± SD from independent duplicate assays.

^a^ Sample was added during viral infection and throughout the incubation thereafter.

^b^ Sample was added immediately after viral infection.

**Fig 2:**
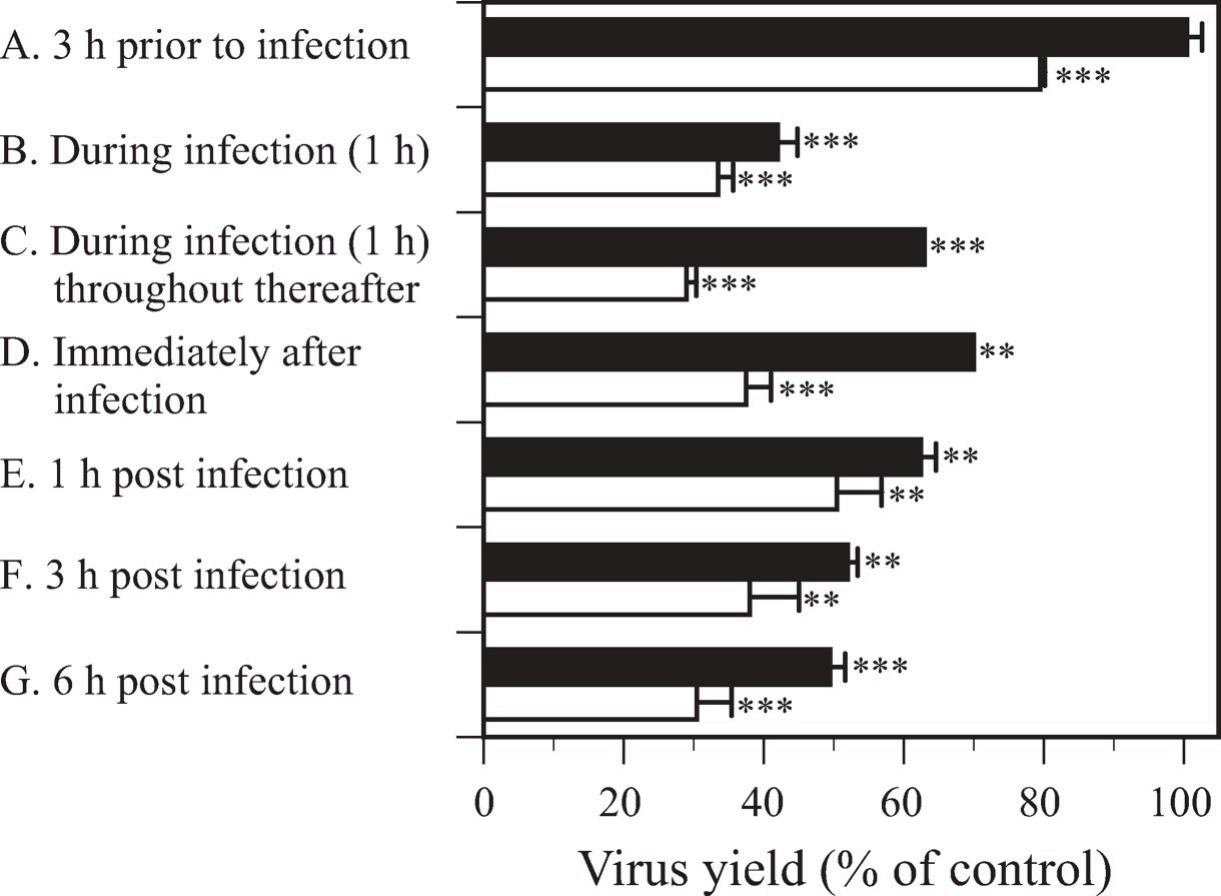
Effects of time of addition of monogalactosyl diacylglyceride (MGDG) on herpes simplex virus (HSV)-2 replication. MGDG at a concentration of 25 μg/ml (closed bar) or 50 μg/ml (open bar) was added to the medium 3 h prior to virus infection (A), during infection for 1 h (B), during infection for 1 h and throughout the incubation thereafter (C), immediately after infection (D), at 1 h post-infection (p.i.) (E), at 3 h p.i. (F), or at 6 h p.i. (G). **p<0.01, and ***p<0.001 vs. the control.

To clarify the most sensitive step of HSV-2 replication in the presence of MGDG, time-of-addition experiments were performed. Pretreating host cells for 3 h prior to virus infection did not significantly inhibit HSV-2 replication (Fig 2). Further, no marked differences in virus replication were observed when MGDG was added during virus infection at 0 h, 1 h, 3 h, or 6 h post-infection. These results show that the host cell-binding step and virus replication in infected cells might not be the antiviral targets of MGDG. Further, the antiviral target of MGDG likely includes events outside the host cell, that is, inactivation of virus particles released from infected cells.

### Antiviral target of MGDG

**Fig 3:**
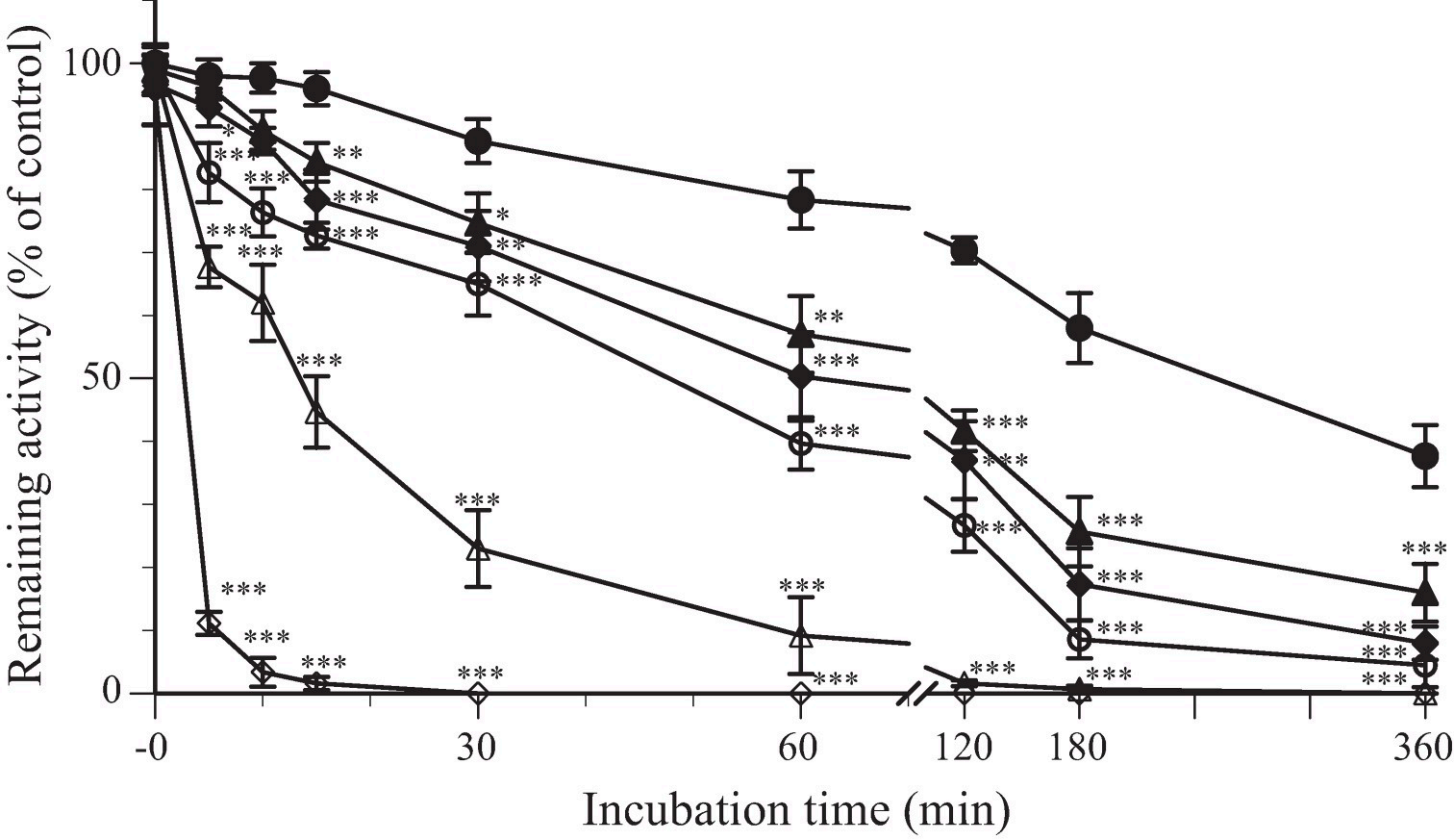
*In vitro* virucidal activity of monogalactosyl diacylglyceride (MGDG) against herpes simplex virus (HSV)-2. HSV-2 (2 × 10^5^ plaque-forming units [PFU]/ml) was mixed with an equal volume of MGDG at specific concentrations (closed circle: 0 μg/ml, closed triangle: 0.01 μg/ml, closed square: 0.1 μg/ml, open circle: 1 μg/ml, open triangle: 10 μg/ml, and open square: 50 μg/ml) and incubated for the indicated time at 37°C. Results are expressed as the percentage of residual infectivity of MGDG-treated virus compared to the percentage of residual infectivity of the mock-treated virus control. Data are means from independent triplicate assays. *<0.05, **p<0.01, and ***p<0.001 vs. the control.

We investigated whether MGDG reduces the infectivity of HSV-2 as a result of interactions between MGDG and virus particles. We assessed MGDG using a virucidal assay based on incubating the virus-compound mixture prior to titrating residual virus infectivity via a plaque assay; results showed that MGDG inactivates HSV-2 in a dose- and time-dependent manner (Fig 3).

To elucidate possible mechanisms underlying the virucidal activity of MGDG, we first assessed the effect of MGDG on virus binding to cell membranes. No plaque was deteced when MGDG-treated HSV-2 was used to infect Vero cells, whereas approximately 100 plaques per well developed in cells infected with mock-related virus (S1 Table). In the second assessment, the effect of MGDG on the sensitivity of host cells to infection was evaluated by pre-treating the cells with MGDG before infection. Around 100 plaques per well developed even after the host cells were pre-treated with 50 μg/ml MGDG for 6 h, approximately the same number that developed in the cell monolayers that were mock treated before infection (S2 Table).

**Fig 4:**
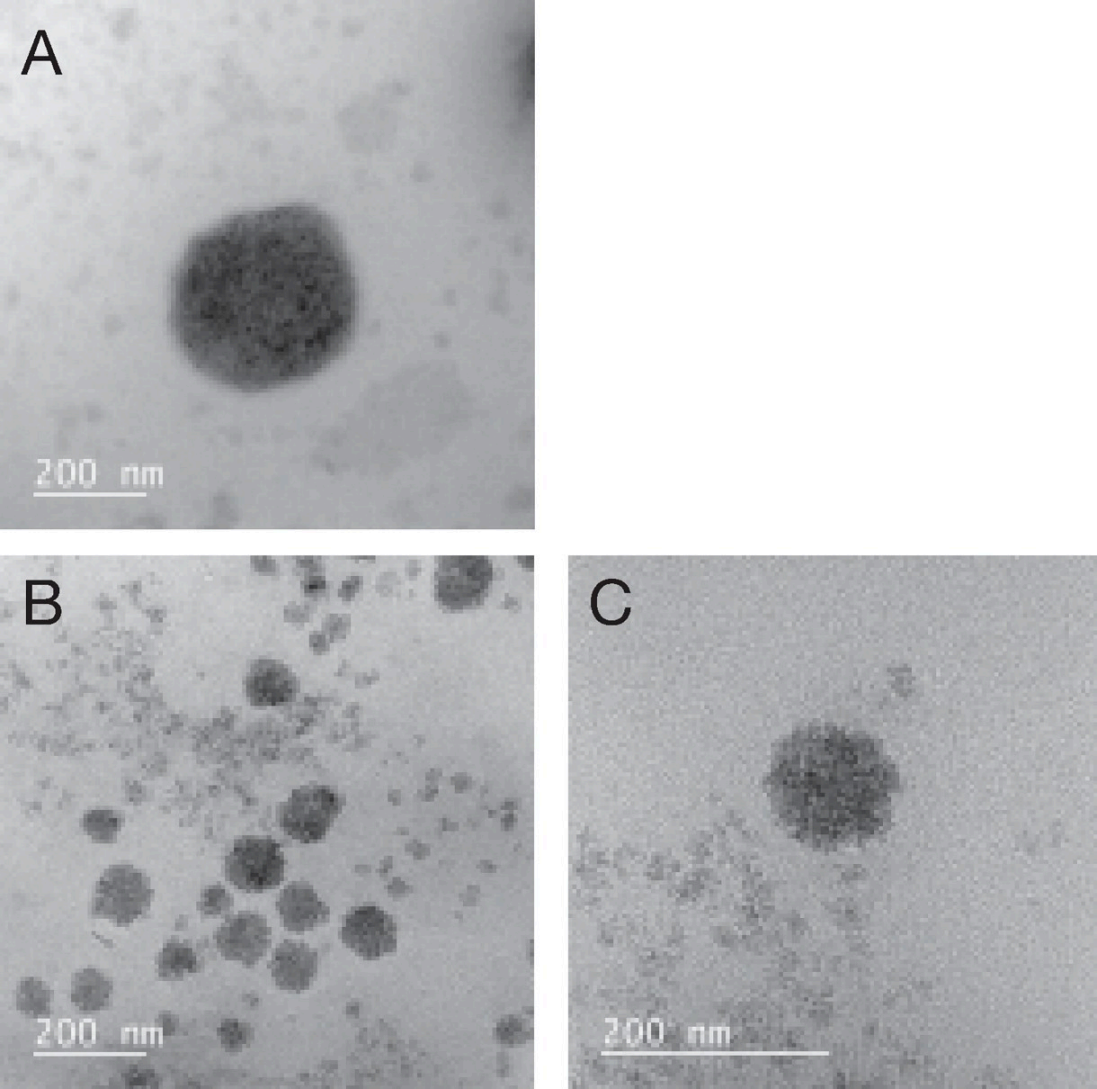
Transmission electron microscopy (TEM) images of herpes simplex virus (HSV)-2 particles subjected to mock or monogalactosyl diacylglyceride (MGDG) treatment. Viral particles released from cells infected with HSV-2 were treated with phosphate-buffered saline (PBS) (A) or MGDG at a concentration of 50 μg/ml (B and C) for 30 min and observed with a JEM ARM 1300 microscope.

We also assessed structural changes in virus particles following treatment with MGDG via TEM. To avoid damage to the structural integrity of viral particles, MGDG-treated viruses were not concentrated or purified by means of ultracentrifugation. TEM images of HSV-2 particles subjected to mock (PBS) or MGDG (50 μg/ml) treatment are shown in Fig 4. We measured a decrease in the particle size of compound-treated material. The virus particle diameter of mock-treated HSV-2 ranged from 272 to 308 nm (n = 5), while the virus particle diameter of MGDG-treated HSV-2 ranged from 66 to 118 nm (n = 20). As the diameter of the HSV capsids is 125 nm, these changes in MGDG-treated virus particles indicated that not only the envelope but also the viral capsids themselves might be affected.

Further, virions released from MGDG-treated cells were detected by negative staining electron microscopy. No morphological disruption of the virions was observed when compared with those released from MGDG-untreated cells (S2 Fig).

### Irreversible virucidal effects of MGDG *in vivo*

**Fig 5:**
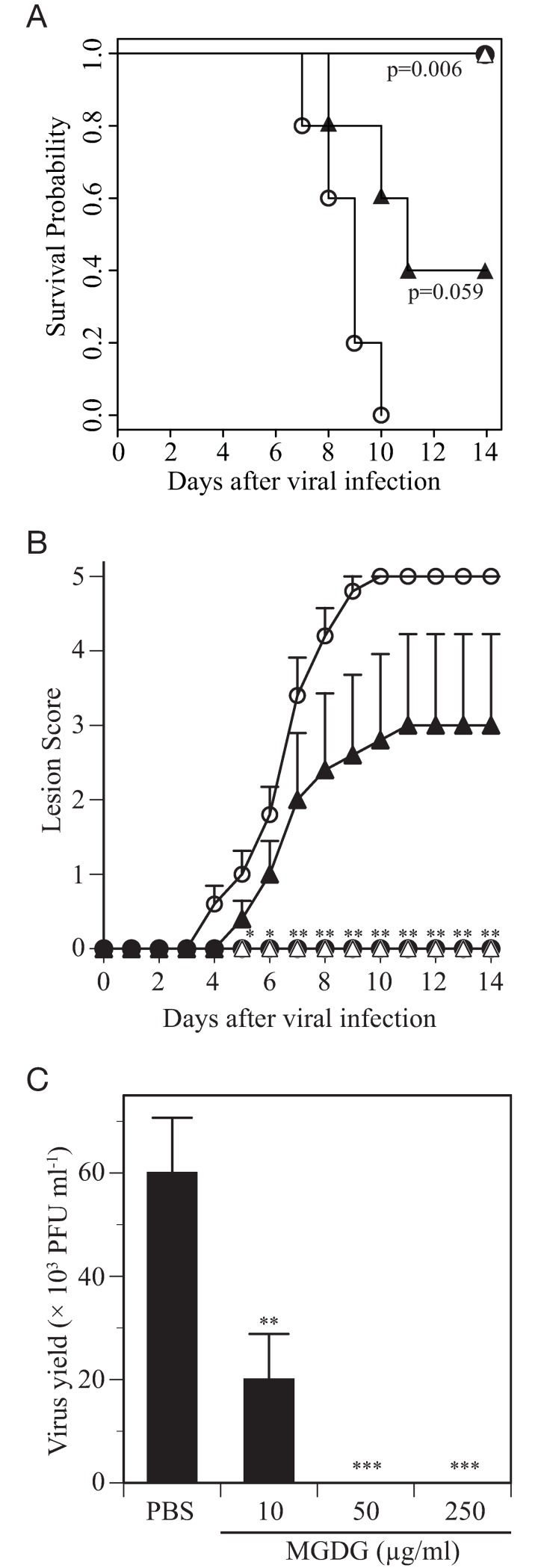
Protection of mice against herpes simplex virus (HSV)-2 infection via short virus treatment with monogalactosyl diacylglyceride (MGDG). HSV-2 (2 × 10^4^ plaque forming units [PFU]/mouse) was treated with PBS (open circle), MGDG at 250 μg/ml (closed circle), 50 μg/ml (open triangle), or 10 μg/ml (closed triangle) for 30 min prior to intravaginal inoculation in mice (n = 5), and animals were monitored for survival (A), lesion scores (B) and infectious virus yields in vaginal lavage fluid sampled at day 3 post-infection (C).

We investigated whether exposing mice to HSV-2 inoculum (2 × 10^4^ PFU/mouse) treated with MGDG for 30 min prior to local administration of the mixture would irreversibly inactivate the virus, thus protecting the animals from genital herpes. All mice in the control group that received the mock-treated virus developed severe disease and found dead (n = 2) or were euthanized (n = 3) by days 7 to 10 post-infection (Fig 5A and 5B). All mice inoculated with the MGDG (50 and 250 μg/ml) survived the experiment (Fig 5A), showing no symptoms of disease (Fig 5B). Mice infected with virus with the lowest concentration of MGDG (10 μg/ml) presented with mild symptoms (Fig 5B) and had a 40% survival rate where two mice were euthanized and one was found dead (Fig 5A). In accord with these results, viral replication in the genital tract was not observed in mice administered virus with the higher concentrations of MGDG (50 and 250 μg/ml), while infectious virus in the vaginal lavage fluid was detected in 4 mice inoculated with the lower concentration (10 μg/ml) of MGDG-treated virus (Fig 5C). These data agree with our results of binding assay of MGDG-treated virus and electron microscopy assessment, which revealed that treatment of HSV-2 virions with MGDG resulted in lysis of virus particles.

### Prophylactic effect of MGDG in mice with genital HSV-2 infection

The virucidal activity of MGDG increases the likelihood of its successful application as a topical drug; therefore, an animal genital herpes model was used to evaluate the activity of intravaginally administered MGDG as a prophylactic measure against genital HSV-2 infection in mice. The doses of MGDG (0.1 mg and 0.5 mg per application) used in the animal experiments were higher than the effective concentrations and were exceeded the CC_50_ in cultured cells.

**Fig 6:**
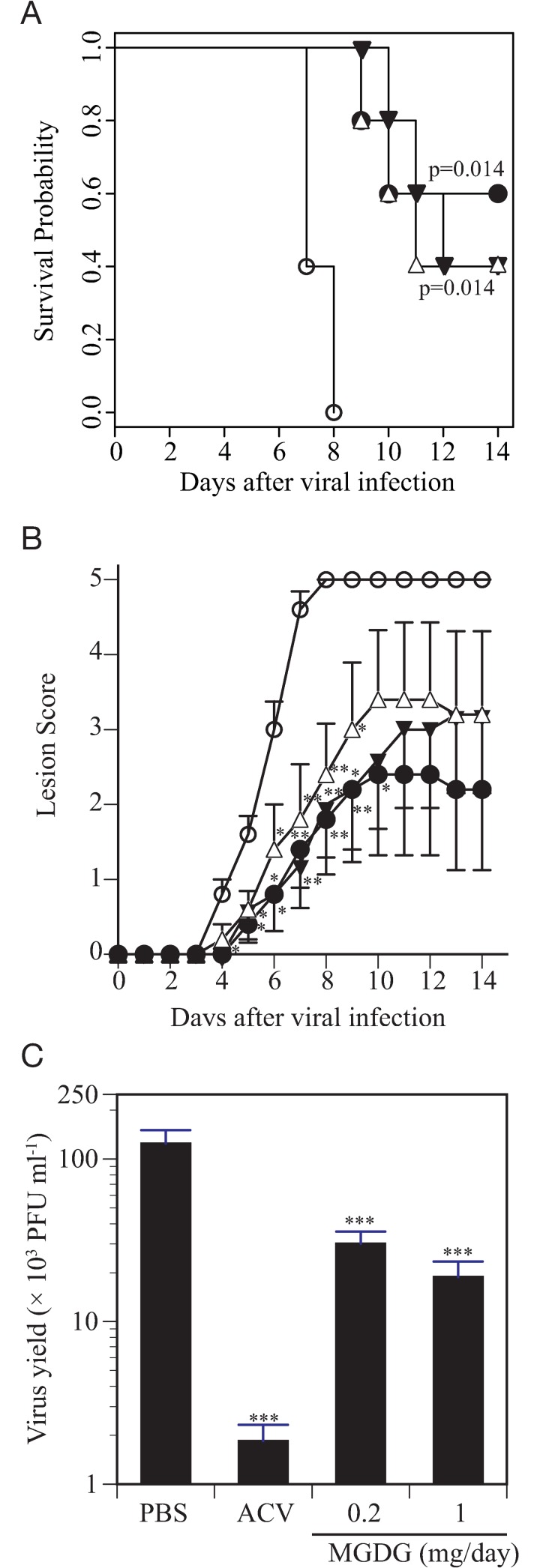
Protection of mice against genital infection with herpes simplex virus (HSV)-2 via prophylactic monogalactosyl diacylglyceride (MGDG) application. MGDG or acyclovir (ACV) was administered intravaginally to mice from 1 h prior to intravaginal HSV-2 (2 × 10^4^ plaque forming units [PFU]/mouse) infection until 7 days post-infection. Animals were monitored for survival probability (A) and lesion scores (B) during 2 weeks of infection. PBS (open circle), ACV at 0.2 mg/day (closed circle), MGDG at 0.2 mg/day (open triangle) or 1 mg/day (closed triangle) were administered to mice (n = 5), respectively. Infectious virus in vaginal lavage fluid sampled at day 3 post-infection was determined using a plaque assay (C). ***p<0.001 vs. the control.

MGDG conferred protection from HSV-2-induced herpetic disease (Fig 6B) and reduced virus replication in the vagina (Fig 6C) in a dose-dependent manner. The survival rate in mice treated with 0.2 mg/day MGDG and 1 mg/day MGDG was 40% and 40%, respectively, where three mice were euthanized and three ones found dead. As such, higher survival rates and delayed death were observed in MGDG-administered mice in comparison with control mice (Fig 6A).

## Discussion

The HSV virion has an outer envelope that consists of a lipid bilayer with viral glycoproteins embedded in it. Mature virions vary in size from 120 nm to as large as 260 nm [20], which approximately agrees with our electron microscopy assessment showing that the diameter of mock-treated HSV-2 particles was around 300 nm (Fig 4).

The first stage of HSV infections involves binding of the viral glycoproteins to a protein on the cell surface. Some existing antiviral agents prevent infection by mimicking cellular proteins, thereby binding to the virus and preventing it from binding with the cells. In the present study, we show that MGDG targets and inactivates HSV-2 viral particles, which may prevent binding, penetration. Treating HSV-2 with MGDG irreversibly decreased infectivity, as virucidal activity was detected even after diluting the mixture of virus and MGDG 100 fold. In addition, the irreversible inactivation of virus was demonstrated via electron microscopy, in which MGDG was observed to cause a physical change in the shape of the virus particle, possibly by damaging the viral envelope (Fig 4). MGDG treatment of virus particles resulted in no plaque formation. These results suggest that MGDG could cause complete lysis of the viral envelope, which is essential for viral attachment to host cells. This phenomenon likely explains the virucidal action of MGDG, which appears to differ from that of ACV, an inhibitor of HSV DNA synthesis.

Pongmuangmul *et al*. suggested that the *in vitro* anti-HSV target of MGDG isolated from the *C*. *nutans* plant is inhibition of late-stage viral replication in infected Vero cells; however, the study did not examine inactivation of HSV by MGDG [18]. Thus, the present study is the first report regarding the *in vitro* virucidal activity of MGDG against HSV and *in vivo* prophylactic effect of topical MGDG application in HSV-inoculated animals.

To date, only a few studies have investigated the fate of galactolipids *in vivo*. Studies in rats suggest that galactolipids are not absorbed intact or as reacylated monoacyl compounds in the blood but are degraded or hydrolyzed in the intestinal tract [21,22]. Therefore, the fate of MGDG in humans should be clarified when determining the bioactivity of the compound after peroral administration. In the present study the doses of MGDG tested in the *in vivo* experiments evaluating its prophylactic effect were higher than the CC_50_.

Taken together, current data demonstrating the antiviral and virucidal properties of MGDG against HSV-2 suggest that MGDG has potential as a novel topical therapeutic for cold sores, viral keratitis, and anogenital herpes. The present study is the first report of the virucidal activity of MGDG isolated from a microalga against HSV-2 and the therapeutic effect of topical application of MGDG to HSV-inoculated mice.

## Supporting information

S1 Fig ^1^H-NMR spectrum of MGDG. (PDF)

S2 Fig TEM images of HSV-2 released from Vero cells treated or untreated with MGDG. Vero cell monolayers were treated with or without 50 μg/ml MGDG at 37°C for 3 h, infected with HSV-2 at 5 PFU/cell and incubated in FBS-free MEM for 20 h. The released viruses were stained with or without potassium Eu-Preyssler-type phosphotungstate.
(PDF)

S1 Table Effect of MGDG on the binding ability of virus particles. (DOCX)

S2 Table Effect of MGDG on host cell membrane evaluated by cell lysis. (DOCX)

S1 File Datasets of Figures. (XLSX)
