# Supplementary figures and images for: In vitro and in vivo anti-herpes simplex virus activity of monogalactosyl diacylglyceride from Coccomyxa sp. KJ (IPOD FERM BP-22254), a green microalga
Source: PLoS One. 2019 Jul 16;14(7):e0219305. doi: 10.1371/journal.pone.0219305 (PMC6634382; doi:10.1371/journal.pone.0219305)

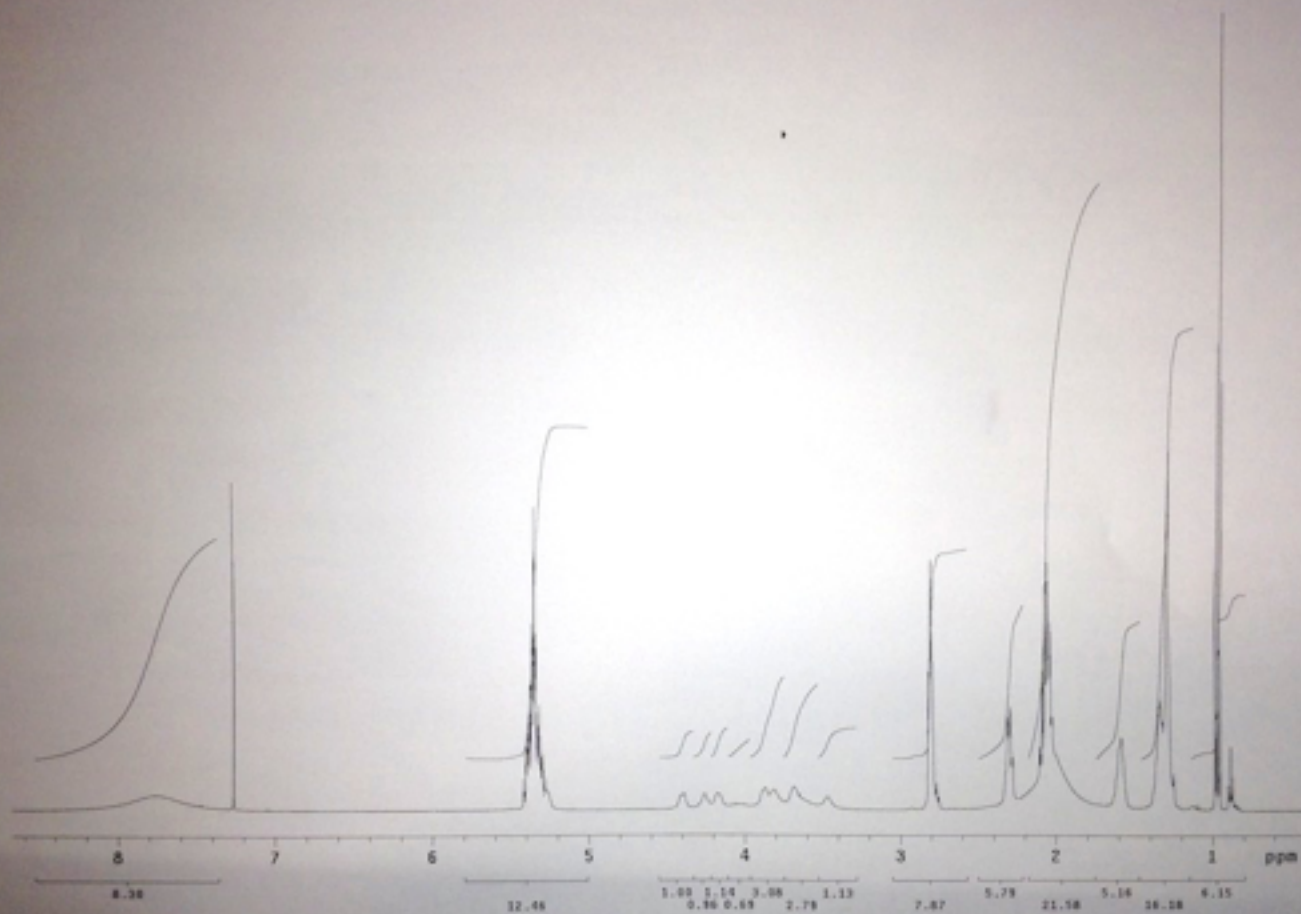

Supplement: S1 Fig — (PDF) [file pone.0219305.s001.pdf]
