# Supplementary material for: In vitro and in vivo anti-herpes simplex virus activity of monogalactosyl diacylglyceride from Coccomyxa sp. KJ (IPOD FERM BP-22254), a green microalga
Source: PLoS One. 2019 Jul 16;14(7):e0219305. doi: 10.1371/journal.pone.0219305 (PMC6634382; doi:10.1371/journal.pone.0219305)
Supplement: S2 Fig — Vero cell monolayers were treated with or without 50 μg/ml MGDG at 37°C for 3 h, infected with HSV-2 at 5 PFU/cell and incubated in FBS-free MEM for 20 h. The released viruses were stained with or without potassium Eu-Preyssler-type phosphotungstate. (PDF) [file pone.0219305.s002.pdf]

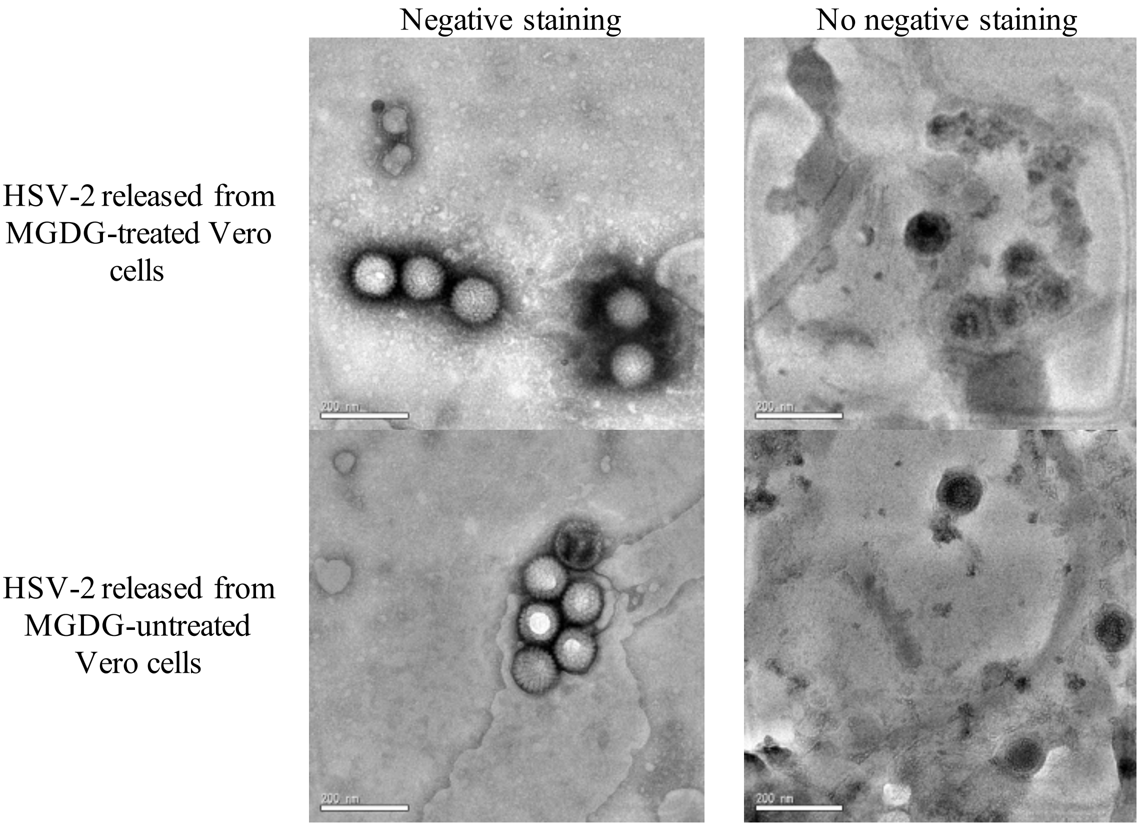
Negative staining
No negative staining
HSV-2 released from MGDG-treated Vero cells
HSV-2 released from MGDG-untreated Vero cells
