# Supplementary material for: In vitro and in vivo anti-herpes simplex virus activity of monogalactosyl diacylglyceride from Coccomyxa sp. KJ (IPOD FERM BP-22254), a green microalga
Source: PLoS One. 2019 Jul 16;14(7):e0219305. doi: 10.1371/journal.pone.0219305 (PMC6634382; doi:10.1371/journal.pone.0219305)
Supplement: S1 Table — (DOCX) [file pone.0219305.s003.docx]

**S1 Table. Effect of MGDG on the binding ability of virus particles.**

|  | MGDG (µg/ml) | |
| --- | --- | --- |
|  | 0 | 50 |
| Plaque number | 103, 97, 106 | 0, 0, 0 |
| Plaque number average | 102 ± 4.6 | 0 |

HSV-2 (100 PFU) was treated with MGDG (0 or 50 µg/ml) at 4˚C for 30 min and added to pre-cooled Vero cell monolayers to be plaque-titrated.
