# Supplementary material for: In vitro and in vivo anti-herpes simplex virus activity of monogalactosyl diacylglyceride from Coccomyxa sp. KJ (IPOD FERM BP-22254), a green microalga
Source: PLoS One. 2019 Jul 16;14(7):e0219305. doi: 10.1371/journal.pone.0219305 (PMC6634382; doi:10.1371/journal.pone.0219305)
Supplement: S2 Table — (DOCX) [file pone.0219305.s004.docx]

**S2 Table. Effect of MGDG on host cell membrane evaluated by cell lysis assay**

|  | Time of treatment of cells with 50 µg/ml MGDG (h) | | | |
| --- | --- | --- | --- | --- |
|  | 0 | 1 | 3 | 6 |
| Plaque number | 106, 109, 101 | 109, 111, 90 | 102, 98, 104 | 100, 112, 104 |
| Plaque number average | 105 ± 4.0 | 106 ±7.0 | 101 ± 3.1 | 105 ± 6.1 |

Vero cell monolayers were incubated at 37˚C for 0, 1, 3 and 6 h in the presence of 50 µg/ml MGDG, and then infected with HSV-2 (100 PFU) to be plaque-titrated.
